# A Preliminary Study: Antibiotic Resistance Patterns of *Escherichia coli* and *Enterococcus* Species from Wildlife Species Subjected to Supplementary Feeding on Various South African Farms

**DOI:** 10.3390/ani10030396

**Published:** 2020-02-28

**Authors:** Michaela Sannettha van den Honert, Pieter Andries Gouws, Louwrens Christiaan Hoffman

**Affiliations:** 1 Centre for Food Safety, Department of Food Science, University of Stellenbosch, Private Bag X1, Matieland 7602, South Africa; 2 Department of Animal Sciences, University of Stellenbosch, Private Bag X1, Matieland 7602, South Africa; louwrens.hoffman@uq.edu.au; 3 Centre for Nutrition and Food Sciences, Queensland Alliance for Agriculture and Food Innovation, The University of Queensland, Coopers Plains, QLD 4108, Australia

**Keywords:** Antimicrobial, bacteria, game, farming

## Abstract

**Simple Summary:**

Supplementary feeding of wildlife allows more opportunity for disease and antibiotic resistant genes to be transferred directly between species due to increased herd density, more frequent direct contact at feeding and water points and increased human contact. The feed itself can also be a direct source of antibiotic compounds and of antibiotic resistant bacteria. This study aimed to determine whether the practice of wildlife supplementary feeding could have an influence on the antibiotic resistance of the bacteria harboured by the supplementary fed wildlife, and thus play a potential role in the dissemination of antibiotic resistance throughout nature. Overall, the *E. coli* and *Enterococcus* isolates from the supplementary fed wildlife were found to be more frequently resistant to the selection of antibiotics than from those which were not supplementary fed. Game farmers should be knowledgeable of the ingredients that are used in the game feed that is used to feed both their livestock and wildlife, as certain feed ingredients, such as antibiotics or bone meal, can have a detrimental effect on health and safety. Game farmers should also be aware that farm history can have an impact on the animals which graze on the pastures with regards to antibiotic resistance transfer.

**Abstract:**

Studies have shown that antibiotic resistance among wild animals is becoming a public health concern, owing to increased contact and co-habitation with domestic animals that, in turn, results in increased human contact, indirectly and directly. This type of farming practice intensifies the likelihood of antibiotic resistant traits in microorganisms transferring between ecosystems which are linked via various transfer vectors, such as rivers and birds. This study aimed to determine whether the practice of wildlife supplementary feeding could have an influence on the antibiotic resistance of the bacteria harboured by the supplementary fed wildlife, and thus play a potential role in the dissemination of antibiotic resistance throughout nature. *Escherichia coli* and *Enterococcus* were isolated from the faeces of various wildlife species from seven different farms across South Africa. The Kirby-Bauer disk diffusion method was used according to the Clinical and Laboratory Standards Institute 2018 guidelines. The *E. coli* (F: 57%; N = 75% susceptible) and *Enterococcus* (F: 67%; N = 78% susceptible) isolates from the supplementary fed (F) wildlife were in general, found to be more frequently resistant to the selection of antibiotics than from those which were not supplementary fed (N), particularly towards tetracycline (*E. coli* F: 56%; N: 71%/*Enterococcus* F: 53%; N: 89% susceptible), ampicillin (F: 82%; N = 95% susceptible) and sulphafurazole (F: 68%; N = 98% susceptible). Interestingly, high resistance towards streptomycin was observed in the bacteria from both the supplementary fed (7% susceptible) and non-supplementary fed (6% susceptible) wildlife isolates. No resistance was found towards chloramphenicol and ceftazidime.

## 1. Introduction

*Escherichia coli* and *Enterococci* are commensal bacteria found in the normal gut flora of animals and are commonly used as indicators of antibiotic resistance due to their ability to easily acquire and transfer antibiotic resistance genes [1].

Food and water sources could be a potential source of antibiotic resistant bacteria as well as act as a selection pressure for the development and spread of antibiotic resistance. In addition, anthropogenic activities such as human encroachment into wildlife habitats, increased transport of wildlife, development of wildlife captive industries and more intensive management of selected wildlife species have been blamed as the likely causes of emerging infectious diseases in humans, as several have originated from wildlife reservoirs [2,3,4].

Due to more intensive wildlife management in South Africa, majority of game farmers provide supplementary feed to their wildlife. Supplementary feeding of wildlife is also a common practice in Europe to alleviate winter mortalities, increase reproductivity and growth and to control the conservation of crops [5,6]. Wildlife supplementary feeding is practiced on 71% of game farms in South Africa, predominantly by specialist game farmers, especially in periods of severe drought [7].

Bekker [7] found that only 13.3% of wildlife feeds that are frequently used by South African game farmers contain antibiotics, according to the packaging label. However, there are various indirect sources of antibiotics which could be added to wildlife feeds that are contained in feed sources such as bone meal, carcass meal and poultry manure [7]. The most commonly used antibiotics in animal feeds in South Africa are macrolides, sulphonamides and tetracyclines, which assist in growth promotion [8]. In wildlife supplementary feeding, the feed is given on a “free-choice” basis by placing the feed at various sites on the farmland at regular intervals. This leads to variable dosing levels of the antibiotics in medicated feeds, possibly promoting the development of drug resistance [9].

It was hypothesised that the bacteria from wildlife which were supplementary fed on a regular basis would be more frequently classified as ‘resistant’ or ‘intermediately resistant’ to the selection of antibiotics than those which were only fed on the land’s natural resources.

## 2. Materials and Methods

### 2.1. Ethics Number

All animals were sampled according to the standard operating procedure approved by the Stellenbosch University Animal Care and Use Committee (ethics number: SU-ACUM14-001SOP).

### 2.2. Study Area and Sample Collection

Supplementary fed and non-supplementary fed blue wildebeest (*Connochaetes taurinus*) (N = 15), African buffalo (*Syncerus caffer*) (N = 19) and impala (*Aepyceros melampus*) (N = 15) faecal samples were collected from seven different farms across South Africa as shown in Table 1. These wild ungulate species are all grazers or mixed grazers-browsers.

South African game farmers most commonly use lucerne/grass as a natural supplement feed for their wildlife. Mineral blocks, commercial feed and self-mixed feeds are also used. The composition of the self-mixed ‘nutrient feed mix’ given to the wildlife on Limpopo farm one is shown in Table 2.

Supplementary fed blue wildebeest samples were collected from farm one in Limpopo. This farm hosts only blue wildebeest which are fed once a day at a single feeding point with multiple troughs containing a nutrient feed mix. The feeding point is rotated around the farm to avoid trampling of the vegetation. The wildebeest are also free to graze on the natural vegetation. All the wildebeest share the same water points which are refilled when necessary. Additional supplementary fed blue wildebeest samples were collected from farm two in Wellington. The wildebeest on this farm are fenced off from all other wildlife species on the farm. They receive supplementary feed when the pastures have become depleted (during summer months) which consisted of a mixture of game pellets, lucerne, oats and molasses. The wildebeest samples that were classified as ‘non-supplementary fed’ were collected from a game reserve in Kwa-Zulu Natal. The game reserve hosts various different wildlife species that have never been supplementary fed or been in contact with others that have been fed and also do not receive any medical treatment. They are free to roam and graze on the open pastures of the reserve, along with the other wildlife species including predators.

Supplementary fed buffalo samples were collected from farm two in Wellington. The buffalo graze on the grass and were occasionally fed a supplementary feed which consisted of game pellets, lucerne, oats and molasses when the pastures had become depleted. The farm which the buffalo roam on is fenced off from all other wildlife and livestock species on the farm. Additional supplementary fed buffalo samples were collected from a different farm in Wellington. The buffalo are fenced off from the other game species and are fed a self-mixed supplementary feed consisting of game pellets, lucerne, oats and molasses when the pastures had become depleted. This farm was previously a sheep farm twenty to thirty years ago. Buffalo which were not fed supplementary feed were collected from the same game reserve in Kwa-Zulu Natal as the wildebeest samples.

Faeces from impala that were supplementary fed was collected from farms one and two in Limpopo. The impala co-graze with other game species on these farms. The game on these farms are given a supplementary feed which is pre-mixed on the farm, its composition is shown in Table 2. The non-supplementary fed impala samples came from the same game reserve in Kwa-Zulu Natal as the buffalo and wildebeest samples.

Wildlife faecal samples were either collected from the ileum of the small intestine from recently slaughtered animals or from the ground shortly after observed deposition. As pertaining to the latter, to avoid sampling from the same animal more than once, faecal samples were selected immediately after observed deposition from the specific animal. Additionally, all samples taken from the same farm were collected on the same day during the same time period to avoid sampling the same animal more than once.

Approximately 20 g of fresh faecal matter was collected off the ground or was taken from the small intestine after evisceration and collected in a sterile, labelled sample container using a new set of gloves for each animal. Some of the samples were taken from the intestine of slaughtered animals, as these animals were used for other research projects.

After sample collection, all faecal samples were stored in a cooler box with ice at ~4 °C and transported to the university’s laboratory freezer within 24 h and stored there at −20 °C until analysis commenced. Metzler-Zebeli et al. [10] found that freezing animal faecal samples at −20 °C had minimal effect (approximately a 3%–6% loss) on the abundance of *Enterococcus* spp. and *Enterobacteriaceae* when compared to sampling directly from fresh faecal samples.

### 2.3. Isolation and Species Confirmation of Bacteria

Faecal samples were defrosted at room temperature for 2 h before analysis commenced. A 10^−1^ dilution of the faecal samples was made by adding 10 g faecal matter to 90 mL Buffered Peptone Water (Merck Biolab, Modderfontein, South Africa). The 10^−1^ faecal dilutions were homogenised using a stomacher for 2 min and incubated at 35 °C for 12–14 h. This incubation resuscitation step assists in recovery of the bacterial cells after frozen storage to allow for more effective isolation. After incubation, 10^−4^ and 10^−5^ serial dilutions of the faecal samples were prepared using Physiological Saline Solution in 10 mL units. These dilutions produced single colonies in the range of 25 to 250 colonies per plate, allowing individual colonies to be easily selected. The pour plate technique was used by pipetting 1 mL from the dilutions onto petri dishes. After this step, selective agar was poured over and swirled in a “figure of 8” motion. Violet Red Bile Dextrose agar (Merck Bioloab, Modderfontein, South Africa) was first used to select for *E. coli* and Baird Parker agar (Oxoid, Hampshire, England) for *Enterococcus* species. *E. coli* characteristic growth on Violet Red Bile Dextrose agar is purple/red colonies surrounded by a halo. Although Baird Parker agar is specifically designed for the isolation of *Staphylococcus aureus*, it is also capable of growing other organisms, such as *Staphylococcus epidermis*, *Enterococcus faecalis* and *Proteus mirabilis*. *Enterococcus* characteristic growth on Baird Parker agar is grey black colonies with no visible zones surrounding the colonies. Vandera et al. [11] successfully used Baird Parker agar as an isolation medium for *Enterococcus*. Once the petri dishes were set, they were inverted and incubated overnight at 35 °C.

Following incubation of the first step of isolation, the streak plate technique was used to streak three random colonies per animal faecal sample onto three selective agar petri dishes. Eosin Methylene Blue agar (Oxoid, Hampshire, England) was used to selectively isolate *E. coli* and Baird Parker agar (Oxoid, Hampshire, England) was again used for *Enterococcus. E. coli* characteristic growth on Eosin Methylene Blue agar is colonies of 2–3mm in diameter with a greenish metallic sheen by reflected light and dark purple centres by transmitted light. The petri dishes were inverted and incubated overnight at 35 °C. A colony from each plate was then transferred onto a Nutrient agar plate (Oxoid, Hampshire, England) and inverted and incubated overnight at 35 °C. These plates were then used to perform the antibiotic susceptibility test on the same day. Gram’s stain and the citrate utilisation test using Simmons Citrate agar (Oxoid, Hampshire, England) was performed on the presumptive *E. coli* isolates to confirm their identity. Gram’s stain, the catalase test and Matrix Assisted Laser Desorption/Ionization-Time of Flight Mass Spectrometry (MALDI-ToF MS) (Bruker, Bremen, Germany) was performed on the presumptive *Enterococcus* isolates to confirm their identity.

The MALDI-ToF MS analysis included a Bacterial Test Standard (Bruker, Bremen, Germany) which was prepared according to manufacturer’s instructions and was applied to the same target plate as the samples. Each sample was tested in triplicate. The MALDI-ToF mass spectra were acquired on an UltrafleXtreme MALDI-ToF/ToF MS instrument (Bruker, Bremen, Germany) using the instrument’s pre-programmed Flex Control 3.0 method MBT_FC.par (Bruker, Bremen, Germany). Spectra were acquired in the linear positive mode within a mass range from 2000 to 20,000 Da. The spectra acquired from each sample were compared to a reference database (Bruker, Bremen, Germany) containing 5627 microorganisms.

### 2.4. Antibiotic Susceptibility Testing

The Kirby-Bauer disk diffusion method was used according to the Clinical and Laboratory Standards Institute (CLSI) 2018 guidelines using Mueller-Hinton agar (Merck Bioloab, Modderfontein, South Africa) and the direct colony suspension method. All animal faecal samples were tested for antibiotic susceptibility in triplicate (each animal was sub-sampled three times). The bacteria were classified as either resistant, intermediately resistant or susceptible, according to the CLSI 2018 zone diameter specifications. Table 3 shows the antibiotic discs (Oxoid, Hampshire, England) used in the analysis and the zone diameter specifications. The discs were placed on inoculated Mueller-Hinton agar plates using an automatic disc dispenser (Oxoid, Hampshire, England).

*E. coli* ATCC 25922 (Thermo Fisher Scientific, Lake Charles, Louisiana) and *S. aureus* ATCC 25923 (Thermo Fisher Scientific, Lake Charles, Louisiana) were used as quality controls and an un-inoculated agar plate was used as a negative control.

Most of the selected antibiotics were chosen based on the fact that they fall into the most commonly used antibiotic classes in the South African agricultural farming sector, as follows: Erythromycin (macrolide), tetracycline (tetracycline), sulphafurazole (sulphonamide), ampicillin and penicillin (penicillin). Additionally, chloramphenicol was chosen as this antibiotic has been banned for use in animal farming and the authors were interested to find out if there would be any resistance to this antibiotic, despite the ban. Furthermore, ceftazidime was selected as this antibiotic gives an indication of the presence of Extended Spectrum Beta-Lactamase (ESBL)-producing bacteria, which is known to be a growing concern to human health as treatment options become limited. Streptomycin was selected as this antibiotic and its accompanying resistance genes are produced by soil microorganisms and thus it was speculated that the ‘native’ streptomycin resistance could be transferred to the grazing wild animals. Lastly, vancomycin was selected as this antibiotic is the ‘drug of last resort’ to treat methicillin-resistant *S. aureus* infections and is a growing concern in the clinical sector. The authors were interested to find out what the level of vancomycin resistance would be outside of a clinical environment, such as wildlife in this case.

Other antibiotics which are recommended for evaluation include but are not limited to, quinolones (ciprofloxacin), aminoglycosides (kanamycin, amikacin, gentamicin, tobramycin), cefoxitin, imipinem, aztreonam, amoxicillin-clavulanate and trimethoprim/sulfamethoxazole for *E. coli* and nitrofurantoin, ciprofloxacin, chloramphenicol and linezolid for *Enterococcus*.

### 2.5. Statistical Analysis

**Figure 1:**
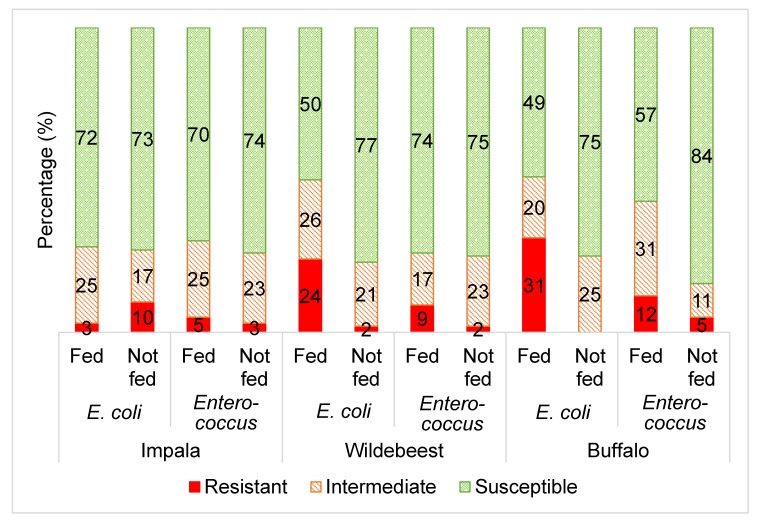
The averaged categorical antibiotic resistance profiles (in percentage) of *E. coli* and *Enterococcus* isolates from supplementary fed (“fed”) wildlife vs. non-fed (“not fed”) wildlife including impala (*E. coli* and *Enterococcus p* > 0.05), wildebeest (*E. coli* and *Enterococcus p* ≤ 0.05) and buffalo (*E. coli* and *Enterococcus p* ≤ 0.05). The percentages of each group (“fed” vs. “not fed”) was calculated by averaging the results from all the antibiotics tested in this study per microorganism (*E. coli*: ampicillin, sulphafurazole, tetracycline, streptomycin, ceftazidime and chloramphenicol; *Enteroccocus*: tetracycline, erythromycin and vancomycin).

**Figure 2:**
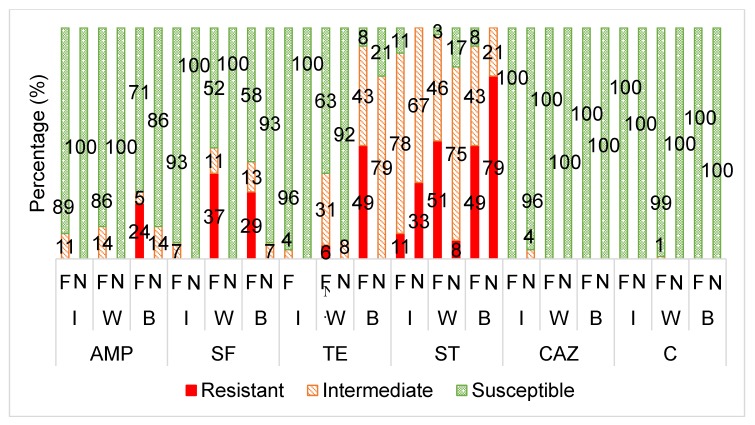
The averaged categorical antibiotic resistance profiles (in percentage) of the *E. coli* isolates from supplementary fed (“F”) wildlife vs. non-supplementary fed (“N”) wildlife against ampicillin (AMP: I, W, B *p* > 0.05), sulphafurazole (SF: I *p* > 0.05; W, B *p* ≤ 0.05), tetracycline (TE: I *p* > 0.05; W, B *p* ≤ 0.05), streptomycin (ST: I *p* > 0.05; W, B *p* ≤ 0.05), ceftazidime (CAZ: I, W, B *p* >0.05) and chloramphenicol (C: I, W, B *p* >0.05). I = impala, W = wildebeest, B = buffalo.

**Figure 3:**
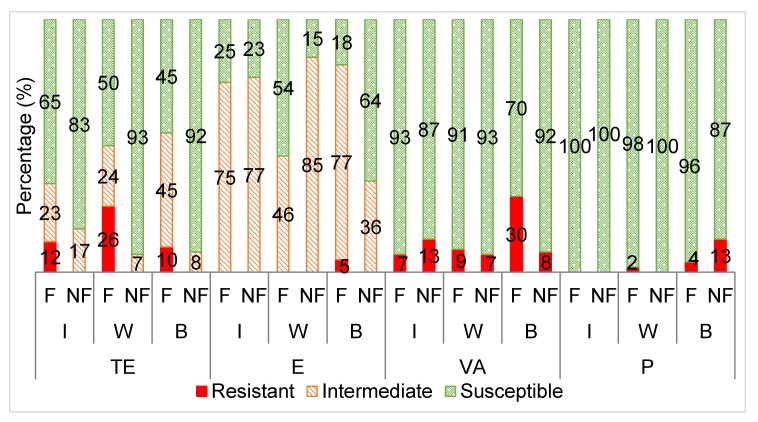
The averaged categorical antibiotic resistance profiles (in percentage) of the *Enterococcus* isolates from supplementary fed (F) wildebeest vs. non- fed (NF) wildebeest against tetracycline (TE: I *p* > 0.05; W, B *p* ≤ 0.05), erythromycin (E: I *p* > 0.05; W, B *p* ≤ 0.05), vancomycin (VA: I, W, B *p* > 0.05) and penicillin (P: I, W, B *p* > 0.05). I = impala, W = wildebeest, B = buffalo.

The frequency of isolates categorised as resistant, intermediately resistant or susceptible to the selection of antibiotics was used to perform the analysis. The analysis was performed separately for *E. coli* and *Enterococcus*. Each animal was sub-sampled three times and these results were included in the overall antibiotic resistance categories of each animal group in order to obtain a more representative sample of the animal groups (see Figure 1). Then each animal group was assigned to either the ‘supplementary fed’ or ‘non supplementary fed’ main effect groups (see Figure 2 and Figure 3). Statistical analysis was performed using Statistica 13.2 software (TIBCO Software, Palo Alto, CA, USA). The data were analysed using one-way analysis of variance (ANOVA). Levene’s test was applied to determine homogeneity of variance. The main effect was the practice of supplementary feeding with animal species as co-variants. Significant results were identified by least significant means (LSM) by using a 95% confidence interval, i.e., a 5% significance level (*p* ≤ 0.05) as a guideline. The significance levels indicated whether or not there were any significant differences in the antibiotic resistant patterns between the two groups.

### 2.6. Antibiotic Resistant Gene Detection

A crude extraction method using lysis buffer and boiling was used to extract DNA from fresh overnight broth cultures of isolated *E. coli*.

The ZymoBiomics DNA kit (Inqaba Biotec, Muckleneuk, South Africa) was used according to the manufacturer’s instructions to extract DNA from fresh overnight broth cultures of isolated *Enterococcus*.

Extracted DNA concentration and quality were determined using a spectrophotometer (Nanodrop-1000) according to the manufacturer’s instructions, using elution buffer as a blank.

Polymerase chain reaction (PCR) was used to detect various antibiotic resistant genes which are commonly associated with phenotypic resistance to the selection of antibiotics. The genes selected and the primers and reaction conditions are listed in Table 4 and Table 5. All reactions were performed in duplicate.

The resistance genes for each corresponding antibiotic was selected based on the genes which have been found to be the most common to each microorganism exhibiting resistance to each antibiotic. In streptomycin-resistant *E. coli,* the *str*A-*str*B gene pair and the *aad*A gene cassette have been found to be the most common streptomycin resistant genes [12,13]. There are six genes that have been identified in tetracycline-resistant *E. coli* strains with the major resistance genes for tetracycline being *tet*A, *tet*B and *tet*C [14]. Sulphonamide resistance is often associated with the *sul*1 and *sul*2 resistance genes in *E. coli* [15]. Researchers have found the *bla*TEM1 gene to be the most common determinant observed in ampicillin-resistant *E. coli* of animal origin [16]. Tetracycline resistance in *Enteroococci* is most commonly due to the presence of the *tet*M gene, but the *tet*K and *tet*L gene are also commonly detected in *S. aureus* isolates [17,18,19]. Vancomycin resistance has been acquired via eight different genes, namely *van*A, *van*B, *van*D, *van*E, *van*L, *van*M and *van*N but the *van*A and *van*B genes are the most common [20,21].

The antibiotic resistance genes not tested in this study but which are recommended for detection include *bla*TEM, *bla*OXA and *amp*C for *E. coli* ampicillin resistance; *flo*R and *cml*A *for E. coli* chloramphenicol resistance; *str*A-*str*B for streptomycin *E. coli* resistance; *sul*3 for sulphafurazole *E. coli* resistance; *bla*CTX-M, *bla*SHV and *bla*OXA for ceftazidime *E. coli* resistance; *erm*A and *erm*B for *Enterococcus* erythromycin resistance and *bla*Z and *pbp*5 for *Enterococcus* (and *Staphylococcus*) penicillin resistance.

The reactions were performed in 25 µL volumes consisting of 1 unit of Ampliqon multiplex TEMPase 2× Master Mix (Ampliqon, Odense, Denmark), 0.2 µM each of forward and reverse primer (Inqaba Biotec, Muckleneuk, South Africa), 1 µL template DNA and the remaining volume distilled nuclease-free water (Inqaba Biotec, Muckleneuk, South Africa).

Gel electrophoresis was performed using 1.2% agarose gel (Lonza SeaKem, Rockland, ME, USA) stained with EZ-Vision^®^ in-gel solution DNA dye (Amresco, Solon, OH, USA). Gels were run for 60–90 min at 85 V. A 100 bp DNA ladder was used (New England BioLabs Inc., Ipswich, MA, USA). Gel visualisation was performed using the Bio-Rad Gel Doc XR+ System (Bio-Rad, Hercules, CA, USA) in combination with Image Lab Software V5.2.1 (Bio-Rad, Hercules, CA, USA).

## 3. Results

### 3.1. Overall Antibiotic Resistance

Figure 1 displays the average antibiotic resistance profiles of *Escherichia coli* and *Enterococcus* from the three wildlife species towards the range of selected antibiotics, comparing the supplementary fed and non-supplementary fed wildlife groups.

### 3.2. Escherichia coli Antibiotic Resistance

Figure 2 displays the antibiotic susceptibility profiles of the *E. coli* isolates from the supplementary fed wildlife (wildebeest, buffalo and impala) versus the non-supplementary fed wildlife species towards each antibiotic.

### 3.3. Enterococcus Antibiotic Resistance

Figure 3 displays the antibiotic susceptibility profiles of the *Enterococcus* isolates from the supplementary fed wildlife (wildebeest, buffalo and impala) versus non-supplementary fed wildlife towards the selected antibiotics.

### 3.4. Antibiotic Resistance Gene Detection

Phenotypic antibiotic resistant patterns were confirmed by detecting commonly-associated antibiotic resistance genes using polymerase chain reaction (PCR). Table 6 and Table 7 display the phenotypic- genotypic antibiotic resistance correlations of the *E.coli* and *Enterococcus* isolates, respectively.

## 4. Discussion

### 4.1. Overall Antibiotic Resistance

Overall, there was a higher frequency (*p* ≤ 0.05) of *E. coli* and *Enterococcus* isolates categorised as resistant to the selected antibiotics from the supplementary fed wildlife compared to the wildlife that were not supplementary fed (Figure 1). This is consistent with the hypothesis that the practice of supplementary wildlife feeding may lead to increased antibiotic resistance of the commensal gut bacteria of the supplementary fed wildlife.

This suggests that either the feed is a direct source of antibiotic resistant bacteria or determinants and/or the actions involved in supplementary feeding may be associated with antibiotic resistance development and transfer. A direct source of antibiotics in animal feed would, of course be the inclusion of antibiotic-based growth/health promoting agents. This would directly result in the development of antibiotic resistance, exemplified by the act of ‘free-choice’ feeding, leading to exposure to sub-inhibitory antibiotic concentrations over prolonged periods. An indirect source of antibiotics and antibiotic resistant bacteria in supplementary feed are rendered animal products such as bone meal, blood meal or fish meal, which are often added to animal feeds as a cheap source of nutrition [7,25,26]. It is suggested that game farmers should become more knowledgeable of the ingredients that are used during the preparation of the feed that is used to feed both their livestock as well as their wildlife, as certain feed ingredients, such as antibiotics, can have a detrimental effect on health and safety.

Furthermore, the practice of supplementary feeding leads to crowding of animals at feeding and water sites, increasing the likelihood of antibiotic resistant determinants transferring between neighbouring animals. The practice of supplementary feeding is also commonly associated with increased human contact, which could also further facilitate the transfer of antibiotic resistant elements to and from the wildlife [27]. Other studies have found that humans act as potential transfer vectors of antibiotic resistant bacteria, as the bacteria from wild animals living in close proximity to human activity were found to be more antibiotic resistant than those from wild animals living in more remote areas [28,29,30,31].

Unlike the wildebeest and buffalo groups, the *E. coli* and *Enterococcus* from the impala groups did not show any significant differences in the frequency of antibiotic resistance between the supplementary fed and non-supplementary fed groups (Figure 1). This seems to be a result of lower antibiotic resistance levels in the impala supplementary fed group, when compared to the wildebeest and buffalo (Figure 2 and Figure 3). This could be attributed to the fact that impala is known to be a species that do not readily take to artificial/supplementary feed.

The grazing/browsing nature of the game species analysed in this study may also play a part in the transfer and development of antibiotic resistance, as the action of grazing allows more direct contact with the soil bacteria that is known to contain naturally produced antibiotic compounds and the accompanying antibiotic resistant genes [32].

### 4.2. Escherichia coli Antibiotic Resistance

Overall, the *E. coli* isolates had the highest frequency of resistance towards streptomycin (38.5%), followed by sulphafurazole (11%), tetracycline (9%), ampicillin (4%) and very low resistance frequency to ceftazidime (0.6%) and chloramphenicol (0.2%). Table 8 displays the relative frequencies of resistance of *E. coli* isolates from various wild animals conducted in other studies. The results from this study fall within the relative frequencies of resistance detected in these other studies.

The *E. coli* isolates from the supplementary fed wildlife had a significantly higher frequency of resistance to streptomycin, sulphafurazole and tetracycline than the isolates from the wildlife that received no supplementary feed (Figure 2).

Tetracycline resistance is commonly found in domesticated farm and food animals (chicken, pig and cattle) [36,37]. Tetracyclines, sulphonamides and streptomycin are antibiotics commonly used for growth promotion in animal feed as they stimulate weight gain [38]. It is possible, though not examined in this study, that the wildlife animal feed contained these antibiotics, either added directly or from indirect sources and hence the significantly higher frequency of antibiotic resistance observed in the bacterial isolates from the supplementary fed wildlife bacteria. In South Africa, it is standard practice for farmers to either mix their own feed on-farm or to buy a mixed feed or concentrate from a commercial animal feed manufacturing company. Irrespective of who mixes the feed, a commercial vitamin and mineral premix is added and it is not uncommon for this premix to contain antibiotic compounds [39]. Unfortunately, this information was not disclosed to the researchers by the farmers/managers and therefore not analysed in this study.

Due to their extensive use in both agricultural and clinical settings, tetracycline, sulphonamide and streptomycin resistance has become widespread and significant in food animals [40,41,42,43]. A similar trend has been observed in other studies where it was noted that tetracycline, sulphafurazole and streptomycin resistance was higher in hospital and farm areas than in pristine/natural areas [43,44].

Resistance to tetracycline in *E. coli* is usually acquired by genes *tet*A-E located on plasmids which encode for efflux pump proteins [14]. Both the *tet*A and *tet*B genes were detected in tetracycline resistant *E. coli* samples (Table 6). Bryan et al. [44] found that 97% of tetracycline resistant *E. coli* harboured at least one *tet* gene from a selection of fourteen known *tet* genes; *tet*A and *tet*B have been the most frequently detected *tet* genes in other studies. Gonçalves et al. [45] also found that the *tet*A and *tet*B genes were the most frequent genes reported in tetracycline *E. coli* isolates from Iberian wolf.

The acquisition of altered target enzymes, which act as competitive inhibitors of dihydropteroate synthetase, known as dihydropteroate synthases, is the most common mechanism with which *E. coli* acquire resistance to sulphonamides [14,46]. There are three genes which encode for three types of these enzymes that have been characterised in Gram negatives, namely *sul*1, *sul*2 and *sul*3. In this study, the *sul*2 gene was the only *sul*-gene detected, the *sul1* gene was not detected. Various other studies detected sul genes in sulphonamide resistant *E. coli* in frequencies of sul2 > sul1 > sul3 [47,48,49,50].

Research has determined that the *str*A-*str*B gene pair and the closely related *aad*A gene cassette are the most common resistant determinates that give *E. coli* resistance to streptomycin [48]. The *aad*A gene cassette, detected in this study, encode for aminoglycoside adenyltransferases which are enzymes that inactivate streptomycin and spectomycin [48]. Some of the *E. coli* isolates which showed intermediate resistance to streptomycin had the *aad*A gene (Table 6). Boerlin et al. [12] found a similar pattern in pigs where the *aad*A gene was detected in streptomycin susceptible isolates, resulting in a low (66%) genotype-phenotype correlation for streptomcyin resistance using the microdilution method and detection of *aad*A *and str*A/*str*B.

The *E. coli* from the non-supplementary fed wildlife only showed notable resistance against streptomycin, similar to that of the supplementary fed wildlife, where a large proportion were intermediately resistant (75%) (Figure 2). Dias et al. [31] also found a high percentage of *E. coli* isolates from wild ungulates to be intermediately resistant to streptomycin. The commensal gut bacteria of the wildlife faecal samples in this study could be viewed as a potential reservoir of streptomycin resistance, where if a selective pressure were applied to the wildlife environments, high levels of ‘complete resistance’ (total non-sensitivity) could emerge.

A study on the antibiotic resistance of soil bacteria revealed that most intrinsically resistant bacteria originate from the soil, where multidrug resistant bacteria are in abundance [36]. This suggests that the streptomycin resistance observed in the bacteria found in the gut of most of the wildlife from this study could have originated or developed due to the intrinsic presence of streptomycin and its accompanying resistant determinants in the soil, produced by organisms such as *Streptomyces griseus* due to the grazing nature of these wildlife species [38].

Beta- lactam antibiotic resistance in *E. coli* is primarily mediated by the production of β-lactamase enzymes which inactivate the antibiotic [51]. Over 200 β-lactamases have been identified, of which the TEM-1, TEM-2 (*bla*TEM gene), CTX-M (*bla*CTX-M gene), SHV-1 (*bla*SHV gene) and CMY-2 (*bla*CMY-2 gene) enzymes are the most common in *E. coli* [51]. Although there was no significant difference in resistance to ampicillin between the two groups, it is interesting to note that only the *E. coli* isolates from the supplementary fed wildlife group showed complete resistance (R) (Figure 2).

Upon further analysis, the overall ampicillin resistance recorded for the supplementary fed wildlife isolates originated from the buffalo samples from one specific farm, Wellington farm 1 (data not shown). These isolates had a higher frequency (*p* ≤ 0.05) of resistance to ampicillin than those from the other wildlife isolates from the other farms. This farm was once a sheep farm about twenty to thirty years ago. Penicillins are the most widely used antibiotic class in sheep farming in the European Union [39]. Data on the types of antibiotics used in livestock production in South Africa are scare [8]. However, Eagar et al. [8] found that macrolides (42.40%), penicillins (10.70%), tetracyclines (16.70%) and sulphonamides (12.40%) were the top three classes of antibiotics purchased in South Africa for use in food animals. The application of antibiotics during the sheep farming period could have altered the soil dynamics by creating an antibiotic selective pressure, encouraging the development of antibiotic resistant bacteria within the soil [40]. Twenty to thirty years later, the antibiotic resistant bacteria and/or resistant determinants still remain in the soil, possibly in an ‘inactive’ state and are transferred to the grazing wildlife. When a selective pressure is applied, such as the antibiotic susceptibility test, the bacteria were found to show resistance against the antibiotic.

The *E. coli* isolates from the buffalo from Wellington farm 1 were also resistant to other antibiotics commonly used in livestock farming, such as tetracycline and sulphonamides (data not shown).

Majority of the *E. coli* isolates were susceptible to chloramphenicol (Figure 2). This is consistent with other studies who had also found negligible or very low resistance levels towards chloramphenicol of bacteria from wild animals, possibly due to the fact that the use of chloramphenicol in food-producing animals has been banned in South Africa and other countries due to its severe side-effects in humans [34,35,52,53].

Furthermore, an insignificant number of *E. coli* isolates were resistant to ceftazidime (Figure 2). Ceftazidime is a clinically-used third generation cephalosporin antibiotic and is used to screen for ESBL-producing bacteria [54]. ESBL-producing bacteria are a great concern to human health as therapeutic treatment of some bacterial infections is largely compromised [55]. Thus, it is suggested that the *E. coli* isolates from the wildlife in this study were not exposed to nearby sources of ESBL-producing bacteria or third generation cephalosporins. This is consistent with other studies which have shown that the majority of ESBL- *E. coli* originate from human clinical settings. However, recently, there has been an increased proportion of ESBL*-E.coli* isolated from community settings [56].

### 4.3. Enterococcus Antibiotic Resistance

Overall, the *Enterococcus* isolates from this study showed the highest resistance towards vancomycin (12%) and tetracycline (8% resistant; 21% intermediately resistant) and low resistance to penicillin (3%) and erythromycin (0.8%; 66% intermediately resistant). Table 9 displays the relative frequencies of antibiotic resistance of *Enterococci* from various wild animals conducted in other studies. The results from this study fall within the relative frequencies of resistance detected in these other studies.

The *Enterococcus* isolates from the supplementary fed wildlife had a significantly higher frequency of resistance to tetracycline than those from the wildlife that received no supplementary feed (Figure 3). This trend was also seen for the *E. coli* isolates and for reasons previously explained. A similar trend has been observed in other studies where it was noted that tetracycline, sulphafurazole and streptomycin resistance was higher in domesticated animals compared to wild animals [58].

There are two main mechanisms of tetracycline resistance that have been documented in *Enterococci* and *Staphylococci* [59,60]. These are, efflux pumps, encoded by *tet*K and *tet*L and production of a ribosomal protection protein, encoded most commonly by *tet*M but also the *tet*O and *tet*S genes [59]. Resistant strains most often carry the *tet*M gene, as also found in this study, and are known to be resistant to all tetracycline drugs [23,45].

Furthermore, a high proportion of the *Enterococcus* isolates from both groups were classified as intermediately resistant to erythromycin, resulting in no significant differences between the two groups. Others have shown that *Enterococcus* from wildlife are most commonly resistant to tetracycline and erythromycin [1,57]. The erythromycin resistance observed in these studies was mostly linked with an erythromycin resistant gene, *erm*B, which is frequently associated with a highly mobile genetic element, Tn*1545*, commonly found in human clinical isolates, although only phenotypic erythromycin resistance was evaluated in this study. This suggests that the erythromycin resistance observed in the wildlife could have been transferred from contact with human sources [1].

The insignificant differences in resistance observed between the supplementary fed and non-supplementary fed wildlife *Enterococcus* isolates towards penicillin can be attributed to low resistance levels in both groups, leading to little variance in the data. Nowakiewicz et al. [39] also detected no resistance to penicillin by *Enterococcus* from wild animals.

Vancomycin resistant *Enterococcus* was detected in both the supplementary fed (average 15%) and non-supplementary fed (average 9%) wildlife groups, leading to no significant differences in vancomycin resistance between the two groups. Vancomycin- resistant (VR) *E. faecalis* is not common; it has only been detected in between 0.1% and 11% of isolates worldwide, including clinical isolates [61]. On the other hand, VR *E. faecium* is more common and is on the rise with occurrences of up to 80% in various clinical studies [20,34]. However, this study did not investigate the antibiotic resistance of *E. faecium* specifically. The CLSI 2018 guidelines recommend that the minimum inhibitory concentration (MIC) test or PCR for detection of the *van*A gene should be performed to determine vancomycin susceptibility as the disc diffusion method often gives false negatives and thus is considered unreliable.

Upon further analysis, the overall vancomycin resistance recorded for the *Enterococcus* isolates from the supplementary fed wildlife isolates originated from the buffalo samples (30%) from one specific farm, Wellington farm 1 (data not shown). This farm was once a sheep farm and thus vancomycin resistant bacteria could have developed during the sheep farming period and could have remained in the soil and carried over to the grazing buffalo, as previously discussed for the *E. coli* isolates from this same farm. Nowakiewicz et al. [39] found that over half of the *Enterococcus* isolates from wild animals were resistant to vancomycin, indicating the possibility of contact between different ecosystems.

Vancomycin resistance during the sheep farming period could have developed due to the possible inclusion of animal by-products in the livestock feed, such as poultry bone meal or poultry bloodmeal. Avoparcin, which has shown to promote cross-resistance to vancomycin, was used in poultry farming as a feed additive in South Africa and Europe until it was banned in the 1990s [45,62,63]. However, a study done twenty years after the ban in 2002 in South Africa showed that 66.6% of *E. coli* isolates from poultry were resistant to avoparcin [64].

Currently, there are nine vancomycin resistance clusters that have been found in *Enterococci*, *van*A, *van*B, v*an*C, *van*D, *van*E, *van*G, *van*L, *van*M and v*an*N [64]. Of these clusters, the *van*A cluster has been the most common resistance gene in vancomycin resistant *Enterococci*, followed by *van*B [63,64]. In this study, the *van*A gene was most commonly detected in the vancomycin resistant isolates. Detection of the *van*A gene in the *Enterococci* from the faeces of the grazing wildlife could have originated from the soil bacteria, as Guardabassi et al. [65] found that the soil contains genes which are nearly identical to the *van*A gene which typically confers clinical resistance to glycopeptides.

### 4.4. Antibiotic Resistance Genes

Most antibiotic resistant genes were detected with a correct phenotypic correlation, with an average of 93%. The remaining 7% were incorrect phenotypic correlations, where the antibiotic resistant gene/s were detected but the sample was phenotypically antibiotic susceptible to the corresponding antibiotic. This could suggest that PCR is a more sensitive method than the disc diffusion method for antibiotic resistance detection. Alternatively, this discrepancy could be due to the fact that the samples used in this study originated from environments of low antibiotic use, possibly resulting in the presence of inactive genes, which have the potential to become active or “switched on” when a selective pressure is applied. These resistant genes could be native to the microorganisms where the resistant gene has a physiological function but is “silent” in the sense of not showing a detectable form of resistance, since their function is to protect the hosts’ own metabolism [66].

Furthermore, it was noted that most samples that were phenotypically intermediately resistant to an antibiotic, were found to be genotypically negative, except for the streptomycin disc diffusion test where the opposite correlation was found.

## 5. Conclusions

The bacteria from the supplementary fed wildlife had higher frequencies of antibiotic resistance than those which were not supplementary fed, particularly towards tetracycline, ampicillin and sulphafurazole. This may suggest that the greater the intervention of human activities on the animals, the greater the opportunity of antibiotic resistance development and transfer of antibiotic resistance elements.

The only similarities in antibiotic resistance patterns between both groups was towards streptomycin, where the *E. coli* from both the supplementary fed and non- supplementary fed wildlife showed significant resistance, suggesting streptomycin resistance could be native to these bacteria. The antibiotic resistance patterns observed in the bacterial isolates from the non-supplementary fed wildlife species could serve as a baseline in future studies for monitoring the influence that various human activities have on the development of antibiotic resistance in wildlife species.

## Figures and Tables

**Table 1:** Details of the samples collected from different wildlife species for this study.

Species	Farm Type	Farm Name	Number of Animal Faecal Samples	Sample Collection Method	Type of Supplementary Feed
Blue wildebeest	Supplementary fed	Wellington farm 2	5	Ground	Game pellets, lucerne, oats and molasses
Blue wildebeest	Supplementary fed	Limpopo farm 1	5	Intestinal	Nutrient feed mix
Blue wildebeest	Not supplementary fed	KwaZulu-Natal	5	Ground	-
African buffalo	Supplementary fed	Wellington farm 1	5	Ground	Lucerne, oat hay and game pellets
African buffalo	Supplementary fed	Wellington farm 2	5	Ground	Game pellets, lucerne, oats and molasses
African buffalo	Supplementary fed	Graaff- Reinet	4	Ground	Hay, lucerne, game pellets
African buffalo	Not supplementary fed	KwaZulu-Natal	5	Ground	-
Impala	Supplementary fed	Limpopo farm 2	5	Intestinal	Nutrient feed mix
Impala	Supplementary fed	Limpopo farm 3	5	Intestinal	Nutrient feed mix
Impala	Not supplementary fed	KwaZulu-Natal	5	Ground	-

**Table 2:** Composition of the nutrient feed mix given to the wildlife from Limpopo farm one.

Ingredient	Percentage (%)
Brewer’s grain	13
Wheat	6
Soya cake	7
Cotton seed cake	5
Maize	16
Lime	1
Trace mineral supplement	1
Salt	<1
Micronutrient pack	<1
Molasses	12
Lucerne	4
Grass	34

**Table 3:** Antibiotic discs selected for testing and their zone diameter specifications (Clinical and Laboratory Standards Institute, 2018).

Antimicrobial Agent	Disk Content	Zone Diameter (Nearest Whole mm)		
		Resistant	Intermediately Resistant	Susceptible
*Escherichia coli*				
Ampicillin	10 μg	≤13	14–16	≥17
Ceftazidime	30 μg	≤17	18–20	≥21
Chloramphenicol	30 μg	≤12	13–17	≥18
Streptomycin	10 μg	≤11	12–14	≥15
Sulphafurazole	10 μg	≤12	13–16	≥17
Tetracycline	300 μg	≤11	12–14	≥15
*Enterococcus*				
Erythromycin	15 μg	≤13	14–22	≥23
Penicillin	10 U	≤28	-	≥29
Tetracycline	30 μg	≤14	15–18	≥19
Vancomycin	30 μg	-	-	≥15

**Table 4:** PCR conditions for detection of resistant genes in *E. coli* isolates.

Antibiotic	Gene	Primers F: 5’-3’ Primers R: 5’-3’	bp	Reaction Conditions	Reference	Positive Control from This Study
**Tetracycline**	*tet*A	F: GGCGGTCTTCTTCATCATGC R: CGGCAGGCAGAGCAAGTAGA	502	15 min initial denaturation at 95 °C followed by 35 cycles of 20 s at 95 °C, 40 s at 66 °C, and 40 s at 72 °C; and a final extension step of 4 min at 72 °C.	Adapted from [12]	*E. coli* CA4c
	*tet*B	F: CATTAATAGGCGCATCGCTG R: TGAAGGTCATCGATAGCAGG	930			*E. coli* CA4c
**Sulphafurazole**	*sul*1	F: CGGCGTGGGCTACCTGAACG R: GCCGATCGCGTGAAGTTCCG	433	15 min initial denaturation at 95 °C followed by 30 cycles of 20 s at 95 °C, 40 s at 66 °C, and 40 s at 72 °C and a final extension step of 4 min at 72 °C.	Adapted from [22]	*E. coli* BB3a
	*sul*2	F: CGGCATCGTCAACATAACCT R: TGTGCGGATGAAGTCAGCTC	721			*E. coli* BB3a
**Ampicillin**	*bla*CMY	F: GACAGCCTCTTTCTCCACA R: TGGACACGAAGGCTACGTA	1000	15 min initial denaturation at 94 °C followed by 30 cycles of 1 min at 94 °C, 1min at 55 °C, and 1 min at 72 °C and a final extension step of 10 min at 72 °C.	[22]	*E. coli* E1B2b
**Streptomycin**	*aad*A	F: GTGGATGGCGGCCTGAAGCC R: AATGCCCAGTCGGCAGCG	525	15 min initial denaturation at 95 °C followed by 35 cycles of 1 min at 94 °C, and 1 min at 60 °C and 1 min at 72 °C and a final extension step of 7 min 72 °C.	Adapted from [12]	*E. coli* E1B1b

**Table 5:** PCR conditions for detection of resistant genes in *Enterococcus* isolates.

Antibiotic	Gene	Primers F: 5’-3’ Primers R: 5’-3’	bp	Reaction Conditions	Reference	Positive Control from This Study
**Tetracycline**	*tet*K	F: GATCAATTGTAGCTTTAGGTGAAGG R: TTTTGTTGATTTACCAGGTACCATT	1515	15 min initial denaturation at 95 °C followed by 30 cycles of 95 °C for 30 s, 62 °C for 1 min and 65 °C for 1 min and a final extension step of 72 °C for 4 min.	Adapted from [23]	*E. faecalis* I1aM
	*tet*L	F: TGGTGGAATGATAGCCCATT R: CAGGAATGACAGCACGCTAA	229			*E. faecalis* I1aM
	*tet*M	F: GTGGACAAAGGTACAACGAG R: CGGTAAAGTTCGTCACACAC	406			*E. faecalis* I1aM
**Vancomycin**	*van*A	F: GGGAAAACGACAATTGC R: GTACAATGCGGCCGTTA	732	15 min initial denaturation at 95 °C followed by 30 cycles of 95 °C for 30 s, 54 °C for 1 min and 72 °C for 1 min and a final extension step of 72 °C for 4 min.	Adapted from [24]	*E. faecalis* S1d
	*van*B	F: ACGGAATGGGAAGCCGA R: TGCACCCGATTTCGTTC	647			*E. faecalis* SB4c

**Table 6:** Correlation between *E. coli* phenotypic antibiotic resistance and PCR results.

Location	Animal	Phenotypic Resistance ^a^	Genotypic Resistance					
			*bla*CMY	*sul*1	*sul*2	*tet*A	*tet*B	*aad*A1
Limpopo farm 2	Wildebeest 1	AMP(S), SF(S), TE(I), ST(R)	-	-	-	-	-	+
Limpopo farm 2	Wildebeest 2	AMP(I), SF(I), TE(I), ST(R)	+	-	-	-	-	+
Limpopo farm 2	Impala 1	AMP(I), SF(I), TE(I), ST(R)	-	-	-	-	-	+
Limpopo farm 1	Impala 1	AMP(R), SF(S), TE(S), ST(S)	+	-	-	-	-	-
Limpopo farm 1	Impala 2	AMP(S), SF(S), TE(S), ST(S)	-	-	-	-	-	-
Wellington farm 1	Buffalo 1	AMP(S), SF(S), TE(S), ST(R)	+	-	-	-	-	+
Wellington farm 1	Buffalo 2	AMP(R), SF(S), TE(R), ST(R)	+	-	-	+	-	+
Wellington farm 1	Buffalo 3	AMP(I), SF(I), TE(I), ST(R)	+	-	-	-	-	+
Wellington farm 1	Buffalo 4	AMP(R), SF(S), TE(R), ST(R)	+	-	-	-	+	+
Wellington farm 1	Buffalo 5	AMP(R), SF(R), TE(S), ST(R)	+	-	+	-	-	+
Wellington farm 2	Buffalo 1	AMP(S), SF(I), TE(I), ST(R)	+	-	-	-	-	+
Wellington farm 2	Buffalo 2	AMP(S), SF(S), TE(S), ST(I)	+	-	-	-	-	+
Wellington farm 2	Wildebeest 1	AMP(S), SF(I), TE(I), ST(I)	-	-	-	-	-	+
Wellington farm 2	Wildebeest 2	AMP(S), SF(I), TE(S), ST(R)	+	-	-	-	-	+
KwaZulu-Natal	Impala 1	AMP(S), SF(S), TE(S), ST(I)	+	-	-	-	-	-

^a^ AMP: ampicillin (*bla*CMY); SF: sulphafurazole (*sul*1, *sul*2); TE: tetracycline (*tet*A, *tet*B); ST: streptomycin (*aad*A1); S, susceptible; I, intermediate; R, resistant.

**Table 7:** Correlation between *Enterococcus* phenotypic antibiotic resistance and PCR results.

Location	Animal	Phenotypic Resistancen ^b^	Genotypic Resistance				
			*tet*L	*tet*K	*tet*M	*van*A	*van*B
Limpopo farm 2	Wildebeest 1	TE(R), VA(S)	-	-	+	+	-
Limpopo farm 2	Wildebeest 2	TE(R), VA(R)	+	-	+	+	-
Limpopo farm 1	Impala 1	TE(S), VA(R)	-	-	-	+	-
Limpopo farm 1	Impala 2	TE(S), VA(R)	-	-	-	+	-
Limpopo farm 1	Impala 3	TE(S), VA(R)	-	-	-	+	-
Wellington farm 1	Buffalo 1	TE(I), VA(R)	-	-	-	+	-
Wellington farm 1	Buffalo 2	TE(I), VA(R)	-	-	-	+	-
Wellington farm 1	Buffalo 3	TE(I), VA(R)	-	-	-	+	-
Wellington farm 1	Buffalo 4	TE(R), VA(R)	-	-	+	+	-
KwaZulu-Natal	Wildebeest 1	TE(S), VA(R)	-	-	-	+	-
KwaZulu-Natal	Buffalo 1	TE(S), VA(S)	-	-	-	-	-

^b^ TE: tetracycline (*tet*L, *tet*K, *tet*M); VA: vancomcyin (*van*A, *van*B); S, susceptible; I, intermediate; R, resistant.

**Table 8:** Antibiotic resistance frequencies (%) of *E. coli* isolated from various wild animals.

Animal	TE	ST	SF	AMP	NA	C	CAZ	Reference
Wild birds	75	75	-	60	33.3	41.7	0	[1]
Small wild mammals	29	7	12	7	-	2	-	[22]
Rodents	1.5	4	-	2	0	0	0	[29]
Wild ungulates	8	4	-	10	0	0	0	[30]
Wild ungulates	-	<5	-	-	-	-	7–53	[31]
Wolf	30	25	-	25	10	5	0	[32]
Buffalo	0–7.7	3.8–17.3	37.5–38.5	-	-	1.9–5.8	-	[33]
Wild Cervids	8.6	22.1	11	-	0	0	-	[34]
Wild animals	34.8	22.3	-	22.3	14.3	6.3	0.9	[35]
Wild ungulates	38.3	48.4	-	-	-	-	-	[36]

TE: tetracycline; ST: streptomycin^;^ SF: sulphafurazole; AMP: ampicillin; NA: nalidixic acid; C: chloramphenicol; CAZ: ceftadizime.

**Table 9:** Antibiotic resistance frequencies (%) of *Enterococcus* spp. isolated from various wild animals.

Animal	TE	E	VA	P	Reference
Wild birds	87.1	80.6	0	-	[1]
Wolf	55	22	-	-	[45]
Wild ungulates	35.6	37	9	-	[38]
Bison	8	4	1	-	[57]
Undomesticated animals	24	13	21	0	[58]

TE: tetracycline; E: erythromycin; VA: vancomycin; P: penicillin.

## Limitations

No analysis was undertaken to compare supplementary feed that included antibiotics with feed which did not include antibiotics. Additionally, the inclusion of antibiotics in the wildlife supplementary feed was not known. Furthermore, this study was limited to only three wildlife species, only nine different antibiotics were tested and not all of the most common antibiotic resistance genes associated with phenotypic antibiotic resistance were tested for in this study.
